# RAB10 promotes breast cancer proliferation migration and invasion predicting a poor prognosis for breast cancer

**DOI:** 10.1038/s41598-023-42434-1

**Published:** 2023-09-14

**Authors:** Jian Zhuo, Jianjun Han, Yanchun Zhao, Ruiying Hao, Chong shen, He Li, Luxian Dai, Ankang Sheng, Hanyu Yao, Xiaohong Yang, Weiguang Liu

**Affiliations:** 1 School of Clinical Medicine, The Hebei University of Engineering, Handan, 056000 Hebei China; 2 Department of Breast Surgery, Affiliated Hospital of Hebei University of Engineering, Handan, 056000 Hebei China; 3 Department of Outpatient, Affiliated Hospital of Hebei University of Engineering, Handan, 056000 Hebei China; 4 Department of Breast Surgery, Yangzhou Maternal and Child Health Hospital Affiliated to Yangzhou University Medica College, Yangzhou, 225007 Jiangsu China

**Keywords:** Molecular biology, Biomarkers, Oncology

## Abstract

RAB10, a member of the small GTPase family, has complex biological functions, but its role in breast cancer (BC) remains unclear. The aim of this study was to investigate the relationship between RAB10's role in BC, its biological functions, and BC prognosis. An online database was used to analyze the correlation between differential expression of RAB10 in BC and prognosis. The results of immunohistochemical assays in clinical cohorts were combined with the database analysis. The chi-square test and COX regression were employed to analyze the correlation between RAB10 and pathological features of BC. MTT, Transwell, and wound healing assays were conducted to detect BC cell proliferation, invasion, and metastatic ability. Bioinformatics techniques were employed to explore the correlation between RAB10 and BC tumor immune cell infiltration, and to speculate the biological function of RAB10 in BC and related signaling pathways. Our findings suggest that RAB10 expression is elevated in BC and is associated with HER2 status, indicating a poor prognosis for BC patients. RAB10 can promote the proliferation, migration, and invasion ability of BC cells in vitro. RAB10 is also associated with BC immune cell infiltration and interacts with multiple signaling pathways. RAB10 is a potential biomarker or molecular target for BC.

## Introduction

Breast cancer (BC) is the most common malignancy in women worldwide^1^. In 2020, for the first time, BC surpassed lung cancer as the most diagnosed malignancy overall. It is also a leading cause of cancer-related deaths among women, accounting for 15.5% of such deaths^2^. BC is classified into subtypes based on estrogen receptor (ER), progesterone receptor (PR), human epidermal growth factor receptor 2 (HER2/ERBB2), and proliferative index (Ki-67) expression. These subtypes have dramatic differences in treatment and prognosis^3^. However, traditional biomarkers for evaluating treatment efficacy and clinical outcomes are inadequate. Discovering new biomarkers to guide staging, determine prognosis, and assess treatment effectiveness is a great challenge in the era of precision medicine^4^.

RAB10 is a member of the small GTPase family, which is the largest branch of the RAS superfamily^5^. The RAB10 gene is located on human chromosome 2p23.3 and consists of 200 amino acids, with a predicted molecular weight of 23 kDa^6^. RAB10 has complex biological functions and is widely expressed. It is involved in polarization transport in polarized cells, neuronal growth, and regulation of extracellular transport^7^. Recent studies have shown that RAB10 participates in the progression of various malignancies, such as liver cancer, cervical cancer, and glioma, through mechanisms such as acting as a target of non-coding RNA, modulating the AMP activated protein kinase (AMPK) signaling pathway, and regulating autophagy^8–11^. In our previous study^12^, we found that RAB10 may be involved in BC progression, but its mechanism and biological role are unclear.

Thus, in this study, we used bioinformatics techniques to analyze the correlation between RAB10 mRNA expression and prognosis in BC and paraneoplastic tissues, and to explore its potential biological functions by immuno-infiltration and enrichment analysis. We silenced the expression of RAB10 in BC cells using lentivirus to investigate its effects on cell proliferation, migration and invasion. Additionally, we explored the correlation between RAB10 expression in BC tissues and clinicopathological features and prognosis by immunohistochemistry in conjunction with clinical cohorts.

## Methods

### Online data acquisition and analyses

RNA sequencing data of BC patients in the study were obtained from the TCGA database (https://cancergenome.nih.gov/) and analyses were performed using R software (v4.1.3). RAB10 RNA expression levels were analyzed using the TNMplot database (https://tnmplot.com/) for pan-cancer analysis and selected RNA-seq data from paired tumors and adjacent normal tissues of BC for differential expression analysis^13^. The UALCAN database (https://ualcan.path.uab.edu/) was used to access the Clinical Proteomic Tumor Analysis Consortium (CPTAC) and the International Cancer Proteogenome Consortium (ICPC) dataset for pan-cancer analysis of RAB10 protein expression and differential expression analysis of BC and normal tissue^14^. Survival analysis using gene chip data was conducted in Kaplan–Meier Plotter (https://kmplot.com/) to plot the 5-year overall survival (OS) and recurrence-free survival (RFS) prognostic model of RAB10 with BC ^15^. Immunohistochemical images of RAB10 in normal breast tissue and BC tissue were obtained using The Human Protein Atlas data (https://www.proteinatlas.org/) as a control.

### Correlation analysis of RAB10 expression and immune infiltration

We analyzed the correlation between RAB10 expression and immune cells in TCGA samples using the "CIBERSORT" algorithm available at https://cibersort.stanford.edu/. We also examined the differences in the level of immune cell infiltration between the high and low RAB10 expression groups. The analysis process utilized the R packages "ggplot" and "ggpubr."

### Enrichment analysis of RAB10 in BC

We divided the samples from TCGA into high and low expression groups based on the median RAB10 expression. Subsequently, we performed differential gene analysis on the two groups, using a threshold of |logFC| > 1 and FDR < 0.05. GSEA GO and GSEA KEGG^16^ enrichment analysis were conducted on the logFC values sorted by *p* value < 0.05 and false discovery rate (FDR) < 0.25 to identify significantly enriched functional or pathway terms. We employed the R package "clusterProfiler" for this analysis.

### BC cell lines

The breast carcinoma cell lines MDA-MB-231, HCC1937 and SK-BR-3 were obtained from the American Type Culture Collection (ATCC). The MDA-MB-231 cells were cultured in RPMI-1640 medium supplemented with 10% fetal bovine serum (FBS) at 37 °C in a humidified 5% CO_2_ atmosphere. HCC1937 and SK-BR-3 cells were cultured in RPMI-1640 medium supplemented with 10% FBS and 0.1 mg/ml human recombinant insulin at 37 °C in a humidified 5% CO_2_ atmosphere.

### Additional description

Gene Chemistry (Shanghai, China) designed and synthesized a lentivirus containing short hairpin RNAs (shRNAs) targeting human RAB10. The RAB10-shRNA sequence used in this study was 5′-GCCTTCAATACTACCTTTATT-3′. MDA-MB-231, HCC1937 and SK-BR-3 cells were transduced with the lentivirus following the manufacturer's instructions. The efficiency of RAB10 knockdown was determined using real-time quantitative fluorescence polymerase chain reaction (PCR), and stable transduced cells were expanded and harvested.

### Quantitative real-time PCR

The RNA extraction from MDA-MB-231, HCC1937 and SK-BR-3 cells was performed using Trizol (Invitrogen), and the resulting RNA was reverse transcribed following the manufacturer's instructions (Invitrogen). The detection of RAB10 was performed through quantitative real-time PCR, using the following primers: RAB10 forward: TTT CAC ACC ATC ACA ACC TCC, reverse: GGT ACA ACT CTT TTG TCG TCC ATA. GAPDH was used as a control, with the following primers: GAPDH forward: TGA CTT CAA CAG CGA CAC CCA, reverse: CAC CCT GTT GCT GTA GCC AAA.

### Western blotting

BC cells were collected and lysed on ice for 30 min using a lysis solution containing 20 mM Tris–HCl pH 7.4, 150 mM NaCl, 1% Triton x-100 and protease inhibitor, and the supernatant was collected by centrifugation at 12,000 r min^−1^ for 10 min at 4 °C with a radius of 10 cm. Total protein concentration was measured using the BCA Protein Assay Kit (Pierce). Nitrocellulose membranes were closed with 5% skim milk (diluted with 1 × PBS) for 2 h at room temperature. Rab10 antibody is then added at 4° overnight. Then 1 × PBS buffer was washed for 50 min, secondary antibody was added for 6 h at room temperature, and after 20 min of washing, enhanced chemiluminescence agent (Amersham) was added to observe the proteins in a dark room, and after 5 min at room temperature, exposure and development were performed.

### MTT assay

MDA-MB-231, HCC1937 and SK-BR-3 cells were seeded in 96-well plates at a density of 1 × 104 cells per well in 100 μl of cell culture medium (RPMI-1640 supplemented with 10% fetal calf serum). Each group was set up with three replicate wells and incubated in a 5% CO_2_ incubator at 37 °C for 2–5 days. After the incubation period, 10 µL of MTT solution (5 mg/mL) was added to each well, and the cells were further incubated for 4 h. The supernatant was then aspirated and discarded after horizontal centrifugation, and 150 µL of dimethyl sulfoxide (DMSO) was added to each well. The plates were shaken at low speed for 10 min before measuring the optical density (OD) at 490 nm using an enzyme marker.

### Wound healing assay

The experiments were conducted following the manufacturer's guidelines. Briefly, cells were seeded in 24-well culture plates at a density of 2.0 × 105 cells per well and incubated at 37 °C in a 5% CO_2_ incubator until they formed a monolayer. Once the cells had formed a monolayer, a scratch was made using a 1000 μL pipette tip, and the cells were washed twice with PBS. The medium was then replaced with RPMI-1640 and the cells were further incubated at 37 °C in a 5% CO_2_ incubator.

### Transwell assay

The Transwell assay was conducted using a 24-well tissue culture plate and a 12-well cell culture plate that included an 8 μm pore size polycarbonate membrane. The cells were counted and adjusted to a concentration of 5 × 104 cells/mL in RPMI-1640 medium without 10% FBS. Next, 200 μL of the cell suspension was added to the upper chamber of the Transwell, while the lower chamber was filled with complete medium containing 10% FBS. The plates were then incubated in a 37 ℃, 5% CO_2_ incubator for 24 h. After incubation, the cells were fixed in a formaldehyde solution for 25 min, stained with 0.1% crystal violet for 20 min, and washed with PBS. Six random fields of view were selected and photographed under an inverted microscope.

### Human specimens

A total of 164 tissue samples of invasive ductal carcinoma of the breast, along with 5 samples of paracancerous tissue, were included in the study. All samples were obtained from patients who had undergone surgical treatment for BC at the Affiliated Hospital of Hebei Engineering University between January 2014 and June 2016 and had been pathologically diagnosed with invasive ductal carcinoma of the breast. Neoadjuvant chemotherapy or any other systemic tumor treatment was not administered before surgery to any of the cases. All patients received systemic treatment after surgery and had follow-up information available for 5 years. This study is a retrospective study and will not have an impact on the treatment of patients. All methods were performed in accordance with the relevant guidelines and regulations. The study and experimental methods were approved by the Ethics Committee of the Affiliated Hospital of Hebei Engineering University and granted exemptions from patient informed consent.

### Immunohistochemistry (IHC)

Paraffin-embedded BC tissue and corresponding paracancerous tissue were sectioned and subjected to dewaxing in xylene, followed by gradient alcohol hydration. The tissue sections were then subjected to microwave heating in sodium citrate buffer (pH 6.0) to boiling for 10 min. Endogenous peroxidase was blocked using 3% hydrogen peroxide for 20 min, and 3% sheep serum was added and incubated at 37 °C for 20 min. Primary antibody was added and incubated overnight at 4 °C, followed by secondary antibody incubation at 37 °C for 30 min. Color development solution was used and hematoxylin re-staining was performed. The slices were dehydrated with gradient alcohol and sealed with neutral resin. Finally, two senior pathologists independently assessed the expression of RAB10 by reading the films under the microscope. The staining intensity was scored as: dark brown = 3, brown = 2, light yellow = 1, and colorless = 0. The percentage of positive cells to total cells was classified as: > 75% = 4, > 50% to 75% = 3, > 25% to 50% = 2, 5%-25% = 1, and < 5% = 0. The final two multiplied scores of 0–6 were considered negative and 6–12 were considered positive.

### Statistical analysis

In this study, *t* tests were conducted to analyze the results of quantitative real-time PCR, MTT and Transwell assays. The correlation between RAB10 and clinicopathological characteristics of BC was evaluated using the one-way χ^2^ test. Kaplan–Meier curves and log-rank tests were used to analyze the correlation of RAB10 with OS and RFS of BC. Additionally, Cox regression univariate and multivariate analyses were performed to estimate the risk ratio (HR) and 95% confidence interval (CI), and the statistical significance was determined using the two-tailed test at *P* < 0.05. All clinical data were analyzed using SPSS 24.0 software (IBM, Chicago, USA).

### Ethics approval and consent to participate

In this study, which involved all BC sample tissues, the Ethics Committee of the Affiliated Hospital of Hebei Engineering University approved the study number 2021[K]019.

## Results

### Aberrant expression of RAB10 mRNA was associated with poor BC prognosis

**Figure 1:**
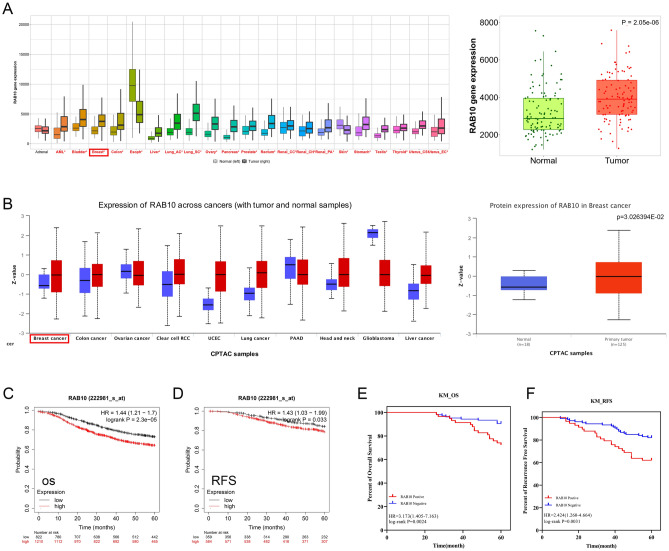
Differential expression of RAB10 in BC cells and prognosis of BC. (**A**) The TNMplot database reveals a significant upregulation of RAB10 mRNA expression in BC (*p* < 0.01). (**B**) The UALCAN database shows a significant upregulation of RAB10 protein in BC (*p* < 0.05). (**C**, **D**) Kaplan–Meier mapping analysis indicates that BC patients in the RAB10 high expression group had worse 5-year overall survival and recurrence-free survival (*p* < 0.05). (**E**, **F**) In the clinical cohort, BC patients with high RAB10 expression had worse overall survival (HR = 3.173, *p* = 0.0024) and recurrence-free survival (HR = 2.424, *p* = 0.0031).

We conducted a pan-cancer analysis of RAB10 mRNA expression using the TNMplot database, which revealed its aberrant expression in a variety of tumors, including BC. Furthermore, we analyzed RNA-seq data from 112 paired BC samples and adjacent paracancerous tissues and found that the mRNA expression level of RAB10 was significantly elevated in BC (*P* = 2.05e−6, Fig. 1A). Subsequently, we used the gene chip dataset in KM plotter to construct survival models of RAB10 and BC over 5 years. The results indicated that patients with high RAB10 mRNA expression had a worse overall survival (HR = 1.43, *p* = 0.033, Fig. 1C) and relapse-free survival (HR = 1.44, *p* = 2.3e−05, Fig. 1D).

### Elevated expression of RAB10 protein in the BC group was associated with poor BC prognosis

**Figure 2:**
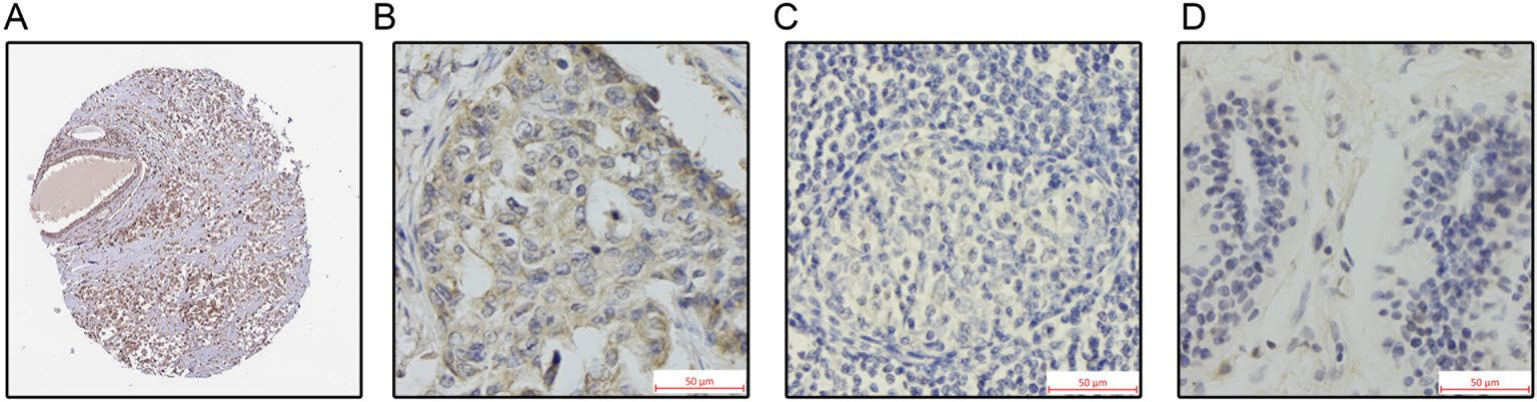
Immunohistochemical staining of RAB10 protein expression in different breast groups. (**A**) Positive expression of RAB10 protein in HPA BC tissue. (**B**) Positive expression of RAB10 protein in clinical cohort BC tissue samples (magnification 400 ×). (**C**) Negative expression of RAB10 protein in clinical cohort BC tissue samples (magnification 400 ×). (**D**) RAB10 protein negative expression in clinical cohort BC paraneoplastic tissues (magnification 400 ×).

The UALCAN database was utilized to analyze RAB10 protein expression levels in 18 normal and 125 BC tissue samples. The results demonstrated significantly higher RAB10 protein expression in BC tissues than in normal tissues (*P* < 0.05, Fig. 1B). We further observed RAB10 expression in BC tissue samples from a clinical cohort through immunohistochemistry staining. RAB10 was predominantly expressed in the cytoplasm of BC cells (Fig. 2B), consistent with HPA database results (Fig. 2A). No RAB10 expression was detected in any of the five BC paracancerous tissues included in the study (Fig. 2D).

RAB10 expression in 164 BC patients was analyzed, with 58 cases showing positive expression and 106 cases showing negative expression (Fig. 2C), resulting in a positive rate of 35%. One-way chi-square tests indicated that RAB10 expression was associated with the tumor grade (*p* = 0.001), molecular staging (*p* = 0.033), and HER2 status (*p* = 0.034) of BC patients (Table 1). After a 60-month follow-up period, 26 patients died and 138 patients survived, with a mean survival time of 57 months, a 5-year relapse-free survival rate of 75.0%, and an overall survival rate of 84.1%. KM curves showed that high RAB10 expression indicated worse OS (HR = 3.173, *p* = 0.0024, Fig. 1E) and RFS (HR = 2.424, *p* = 0.0031, Fig. 1F) in BC patients, consistent with the results from the Kaplan Meier-plotter database. Univariate analysis using the COX regression model indicated that tumor grading, TNM stage, T stage, molecular subtype, and RAB10 status were factors affecting RFS, while TNM stage, T stage, molecular subtype, and RAB10 status were factors affecting OS (Table 2). Multifactorial analysis using the COX regression model indicated that PTNM stage, tumor subtype, and RAB10 status were risk factors for RFS and OS (Table 3).

**Table 1:** The association with RAB10 and clinicopathological factors in invasive BC (N = 164).

Clinicopathologic characteristics	N	RAB10(+)	RAB10(−)	χ^2^	*P*
Nationality					
Han	164	58	106		
Year					
> 60	67	25	42	0.188	0.665
< 60	97	33	64		
Grading					
1	24	3	21	14.993	**0.001**
2	48	11	37		
3	92	44	48		
PTNM stage					
I	42	12	30	2.329	0.312
II	89	31	58		
III	33	15	18		
T stage					
T1	56	16	40	4.025	0.259
T2	80	28	52		
T3	23	11	12		
T4	5	3	2		
N stage					
N0	92	30	62	1.936	0.586
N1	54	20	34		
N2	17	8	9		
N3	1	0	1		
Subtype					
LA	42	11	31	8.713	**0.033**
LB	47	15	32		
HER2+	48	25	23		
TN	27	7	20		
ER status					
Positive	85	25	60	2.737	0.098
Negative	79	33	46		
PR status					
Positive	78	22	56	3.337	0.068
Negative	86	36	50		
HER2 status					
Positive	95	40	55	4.487	**0.034**
Negative	69	18	51		

Significant values are in bold.

**Table 2:** Univariate COX regression analysis of RFS, OS and clinicopathological characteristics of RAB10 (N = 164).

Factor	Recurrence free survival				Overall survival			
	B	*P*	HR	95% CI	B	*P*	HR	95% CI
Year	− 0.207	0.523	0.813	0.431–1.535	− 0.455	0.285	0.635	0.276–1.460
Grading		**0.021**				0.083		
2	0.761	0.336	2.140	0.455–10.080	0.970	0.376	2.638	0.308–22.580
3	1.559	**0.033**	4.752	1.137–19.862	1.763	0.085	5.830	0.782–43.444
PTNM stage		** < 0.001**				**0.002**		
II	0.537	0.292	1.710	0.631–4.635	0.211	0.721	1.235	0.387–3.939
III	1.952	** < 0.001**	7.040	2.623–18.898	1.549	**0.007**	4.707	1.517–14.608
T stage		** < 0.001**				**0.003**		
T2	0.785	0.074	2.193	0.927–5.187	0.493	0.360	1.638	0.569–4.715
T3	1.413	**0.005**	4.108	1.529–11.038	1.426	**0.015**	4.162	1.320–13.123
T4	3.001	** < 0.001**	20.107	6.273–64.451	2.354	**0.001**	10.527	2.506–44.219
N stage		0.103				0.417		
N1	0.454	0.194	1.575	0.794–3.126	0.701	0.093	2.016	0.889–4.570
N2	1.042	**0.014**	2.836	1.232–6.528	0.452	0.488	1.571	0.438–5.631
N3	− 9.821	0.973	0.000	0.000–8.224E+245	− 9.750	0.979	0.000	0.000-
Subtype		**0.018**				**0.042**		
LB	0.030	0.951	1.030	0.398–2.671	0.145	0.828	1.156	0.311–4.307
HER2+	0.251	0.589	1.285	0.517–3.195	0.611	0.319	1.841	0.554–6.116
TN	1.152	**0.010**	3.166	1.311–7.644	1.419	**0.018**	4.131	1.272–13.421
ER status	− 0.510	0.108	0.601	0.323–1.118	− 0.773	0.061	0.462	0.206–1.036
PR status	− 0.788	**0.019**	0.455	0.235–0.878	− 1.202	**0.010**	0.301	0.121–0.749
HER2 status	− 0.405	0.195	0.667	0.362–1.231	− 0.338	0.388	0.713	0.330–1.538
RAB10	0.887	**0.005**	2.428	1.313–4.489	1.156	**0.004**	3.179	1.442–7.008

Significant values are in bold.

**Table 3:** Multi-factor COX regression analysis of RFS, OS and clinicopathological characteristics of RAB10（N=164）

Factor	Recurrence free survival				Overall survival			
	B	P	HR	95% CI	B	P	HR	95% CI
Grading		0.206						
2	0.761	0.340	2.141	0.448–10.228				
3	1.266	0.101	3.546	0.783–16.064				
PTNM stage		**0.002**				**0.050**		
II	0.319	0.723	1.375	0.236–8.025	– 0.035	0.977	0.977	0.091–10.263
III	1.893	**0.042**	6.641	1.067–41.331	1.419	0.258	4.132	0.354–48.259
T stage		0.064				0.328		
T2	0.256	0.740	1.292	0.284–5.882	0.357	0.743	1.429	0.169–12.079
T3	1.047	0.204	2.851	0.565–14.373	1.390	0.222	4014	0.431–37.414
T4	2.206	**0.028**	9.076	1.276–64.573	1.668	0.208	5.300	0.396–70.974
Subtype		** < 0.001**				** < 0.001**		
LB	− 0.540	0.313	0.583	0.204–1.665	− 0.006	0.994	0.994	0.261–3.792
HER2+	− 0.981	0.097	0.375	0.118–1.194	− 0.330	0.644	0.719	0.178–2.910
TN	1.552	**0.004**	4..720	1.627–13.693	2.159	**0.002**	8.660	2.279–32.909
RAB10	0.978	**0.006**	2.659	1.327–5.328	1.330	**0.002**	3.780	1.621–8.812

Significant values are in bold.

### RAB10 promotes proliferation, migration and invasion of BC cells in vitro

**Figure 3:**
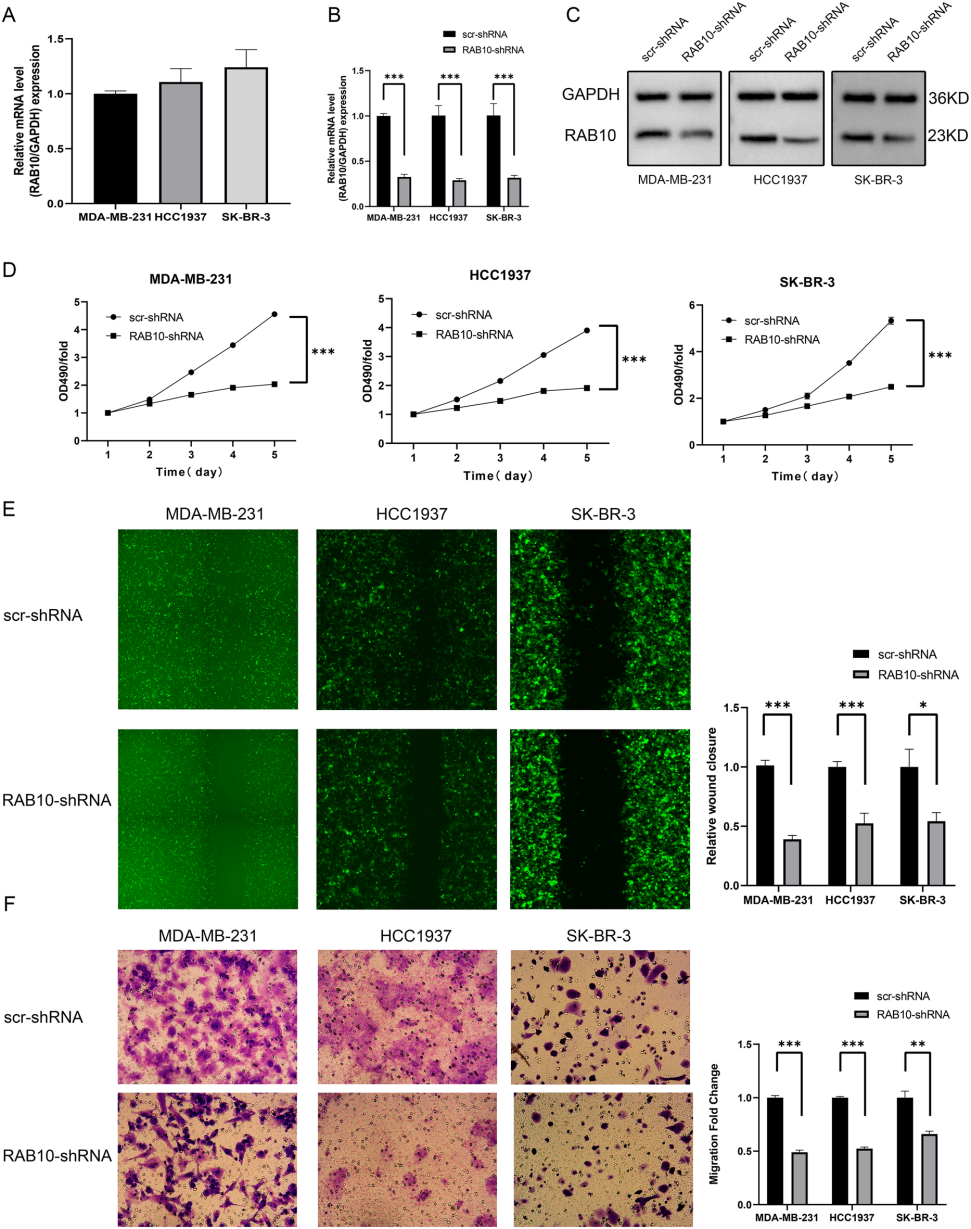
Effects of RAB10 on proliferation, migration and invasion of BC cells in vitro. (**A**) RAB10 mRNA expression levels were measured in two BC cell lines using quantitative real-time PCR. (**B**) RAB10-shRNA infection of MDA-MB-231, HCC1937 and SK-BR-3 cells was detected using quantitative real-time PCR. (**C**) RAB10-shRNA infection of HCC1937 and MDA-MB-231 cells was detected using western blotting. (**D**) The MTT assay was used to assess cell proliferation in MDA-MB-231, HCC1937 and SK-BR-3 cells (*p* < 0.05). (**E**) The wound healing assay was performed to assess cell migration in MDA-MB-231, HCC1937 and SK-BR-3 cells (*p* < 0.05). (**F**) The transwell assay was performed to assess cell invasion in MDA-MB-231, HCC1937 and SK-BR-3 cells (*p* < 0.05).

RAB10 expression levels in MDA-MB-231, HCC1937 and SK-BR-3 cells were examined using quantitative real-time PCR technique (Fig. 3A), followed by knockdown studies.

In this study, shRNA was used to knock down RAB10 expression in MDA-MB-231, HCC1937 and SK-BR-3 cells, while the lentivirus-infected group that carried scrambled siRNA was used as a control. The mRNA expression level of RAB10 was measured after three days of culture, and the results showed a significant reduction in RAB10 expression in knockdown cells compared to control cells (Fig. 3B). Meanwhile, we examined the expression level of RAB10 protein, and the results showed that RAB10 protein expression was also significantly downregulated in BC cells of the RAB10 knockdown group (Fig. 3C).

The proliferation rate of MDA-MB-231, HCC1937 and SK-BR-3 cells was determined using MTT assay after RAB10 knockdown with shRNA or control scr-shRNA. The results showed a significant decrease in cell proliferation rate and cell number after five days in the RAB10 knockdown group compared to the control group (Fig. 3D).

The effect of RAB10 on the migration and metastatic ability of BC cells was evaluated using wound healing and transwell assays. The results showed that the migration and invasion of RAB10-downregulated MDA-MB-231, HCC1937 and SK-BR-3 cells were significantly slower compared to the control group infected with scr-shRNA (Fig. 3E,F), suggesting that RAB10 promotes the migration and invasion of BC cells in vitro.

### Correlation of RAB10 with immune cell infiltration

**Figure 4:**
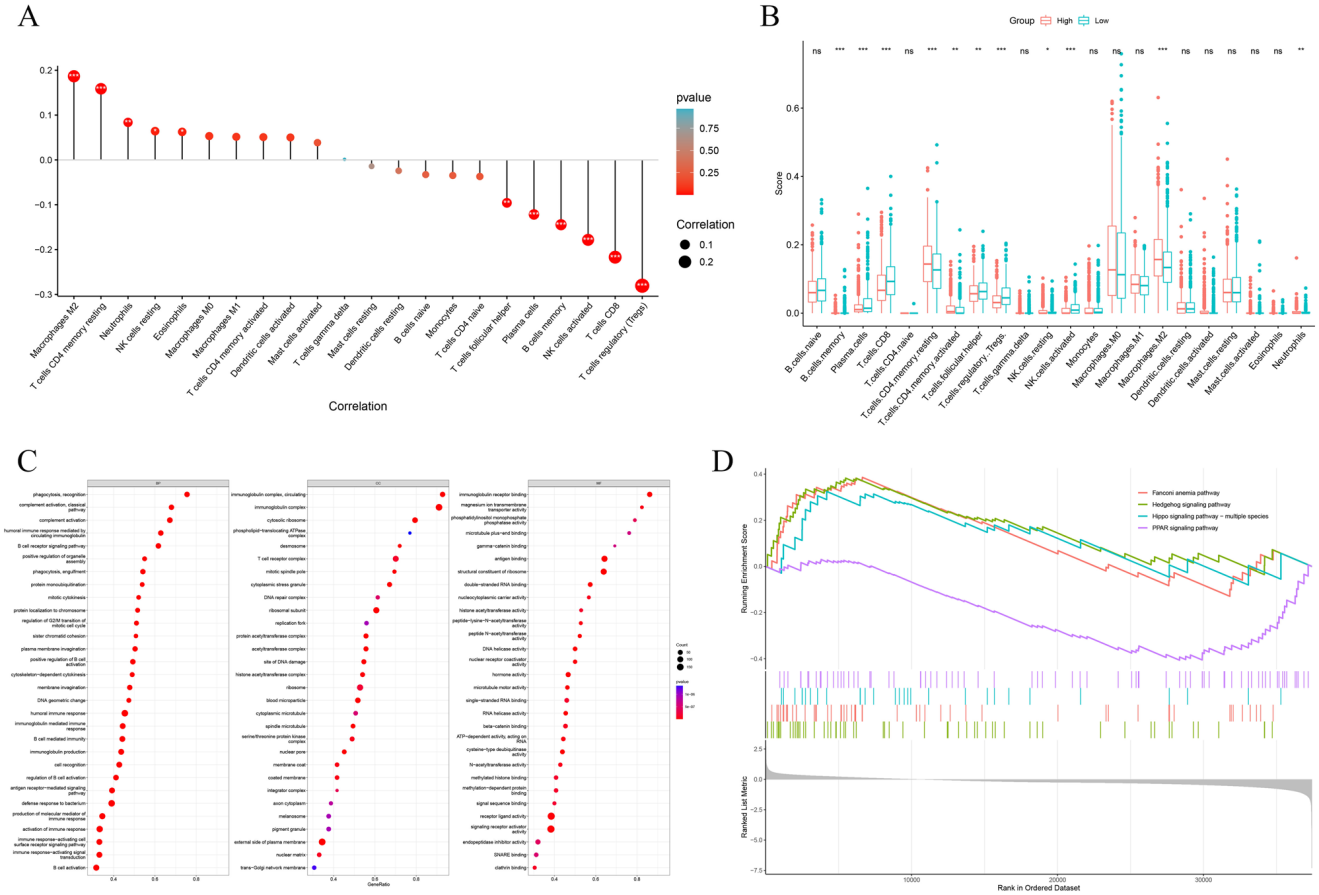
Correlation analysis and biological function prediction of RAB10 with BC tumor immune cell infiltration. (**A**) RAB10 expression was positively correlated with macrophages M2, T cells CD4 memory resting, neutrophils, NK cells resting and eosinophils, but negatively correlated with Tregs, T cells CD8, NK cells activated, B cells activated, plasma cells and T cells follicular helper. (**B**) In the RAB10 high expression group, infiltration levels of macrophages M2, T cells CD4 memory resting, and T cells CD4 memory activated were upregulated, while infiltration levels of B cells memory, plasma cells, T cells CD8, T cells follicular helper, Tregs, NK cells activated, and neutrophils were downregulated. (**C**) Gene set enrichment analysis (GSEA) showed that the majority of RAB10 biological functions were enriched in immune response. (**D**) GSEA KEGG analysis showed that the Hedgehog signaling pathway, Fanconi anemia pathway, Hippo signaling pathway, and PPAR signaling pathway were heavily enriched.

The "CIBERSORT" analysis revealed a positive correlation between RAB10 expression and immune cell infiltration of Macrophages M2, T cells CD4 memory resetting, Neutrophils, NK cells resetting, and Eosinophels, while a negative correlation was observed with Tregs, T cells CD8, NK cells activated, B cells activated, Plasma cells, and T cells follicular helper (Fig. 4A). Furthermore, based on the median RAB10 expression grouping, the RAB10 high expression group showed upregulation in the infiltration levels of Macrophages M2, T cells CD4 memory resting, and T cells CD4 memory activated, while the infiltration levels of B cells memory, Plasma cells, T cells CD8, T cells follicular helper, Tregs, NK cells activated, and Neutrophils were down-regulated (Fig. 4B).

### Biological function and signaling pathway prediction of RAB10 in BC

Based on TCGA RAB10 mRNA expression data, we performed GSEA enrichment analysis on the differential genes in the RAB10 high and low expression groups sorted by logFC. GSEA GO analysis revealed that RAB10 BP is associated with phagocytosis recognition and the classical pathway of complement activation; MF is associated with binding to immunoglobulin receptors; and CC is located in circulating immunoglobulin complex, immunoglobulin complex, and cytosolic ribosome (Fig. 4C). Furthermore, GSEA KEGG analysis demonstrated that RAB10 interacts with the Hedgehog signaling pathway, Fanconi anemia pathway, Hippo signaling pathway (multiple species), and PPAR signaling pathway (Fig. 4D).

## Discussion

We observed abnormal upregulation of RAB10 mRNA and protein in BC tissues through a network cohort and constructed RFS and OS curves with BC patients over 5 years, showing that abnormal expression of RAB10 is associated with poor prognosis in BC.

Considering the potential bias between network cohort and clinical cohort data, we conducted immunohistochemical staining of 164 BC tissue samples. Our results demonstrated an association between RAB10 expression and Grading, Subtype, and HER2 status in BC patients. COX univariate and multifactorial regression analyses also demonstrated that RAB10 is a risk factor for poor prognosis in BC. However, given that our study is retrospective, potential selection bias may exist due to the small sample size of N3 staging in the cohort, leading to the need for further justification of the findings regarding N staging in the COX regression. Our results suggest that abnormal expression of RAB10 is associated with poor prognosis in BC, especially in HER2+ BC.

HER2 is a transmembrane growth factor receptor protein that belongs to type I, and it is found localized to the cell membrane, when HER2 is activated, it triggers dimerization in the intracellular structural domain, leading to the phosphorylation of specific tyrosine residues. This activation subsequently initiates a series of signaling cascades, including the PI3K-Akt and Ras/Raf/MAPK pathways. The endocytosis of the complex formed by the EGFR receptor and its corresponding ligands plays a significant role in terminating the process^17,18^. Rab10 is able to participate in both extracellular and endocytic pathways and can lead to dysregulation of biological functions by altering the expression of some proteins on the plasma membrane^7,19^. HER2 overexpression is commonly associated with poor prognosis and chemotherapy response in BC ^20^. A previous study has shown that RAB10 upregulation in extracellular vesicles from HER2-positive BC cells is associated with trastuzumab resistance, although the mechanism remains unclear^21^. Rab10 is also involved in PI3K-Akt and MAPK signaling pathways^22^. Therefore, we speculate that RAB10 may be involved in the progression of HER2+ BC by altering HER2 expression on the cell membrane or by participating in HER2-mediated downstream signaling pathways.

Next, we examined the expression level of RAB10 in three BC cell lines, and the results showed that the expression level of RAB10 was higher in HER2 + -positive BC cells than in MDA-MB-231 and HCC1937 cells. Further functional experiments showed that down-regulation of RAB10 expression significantly affected BC cell proliferation, migration and invasion. While our online database of large samples and immunohistochemical results in clinical cohorts did not compare RAB10 mRNA and protein expression in BC cells with normal breast cells, further proof is still required to determine the relevant mechanisms by which RAB10 affects functional changes in BC cells.

During the immunohistochemical analysis of BC tissue samples, pathologists observed the expression of RAB10 in infiltrating immune cells around the BC. Therefore, we employed bioinformatics techniques to investigate the relationship between RAB10 and immune cell infiltration in BC. Our findings revealed a positive correlation between RAB10 expression and macrophage M2 memory, which was upregulated in the RAB10 high-expression group. In contrast, NK cells and T cell CD8 exhibited a negative correlation with RAB10 expression and were down-regulated in the RAB10 high-expression group. BC tumor microenvironment (TME) is composed of a diverse population of innate and adaptive immune system cells, and HER2-positive tumors with high genomic instability and tumor mutation burden (TMB) exhibit the highest levels of immune infiltration, which in turn is associated with a high level of tumor-infiltrating lymphocytes (TILs). The immune TME has been identified as a potential prognostic factor in HER2-positive BC ^23,24^. Increased macrophage M2 predicts the tumor's ability to progress through angiogenesis, tissue remodeling, and adaptive immunosuppression^25^; T-cell CD8 acts as an effector cell against tumor cells, whereas NK cell expression is closely associated with pathological complete response of HER2+ BC^26,27^. Prior research has demonstrated RAB10's involvement in Toll-like receptors involved in the innate immune response^28,29^. RAB10 specifically regulates macropinocytosis, a specialized form of endocytosis, thus activating the PI3K-Akt pathway and promoting the immune response of phagocytes^30^. RAB10 can cross-react specifically with antibodies, leading to increased cytokine production and promoting the phagocytic capacity of monocytes^31^. In immune cells, phosphorylation levels of RAB10 correlate with plasma inflammatory factor levels^32^. Based on our previous findings^12^, we have observed that FAM49B has the ability to regulate the RAB10/TLR4 signaling pathway, which plays a crucial role in breast cancer progression. However, the association between RAB10 and immune infiltrating cells in breast cancer remains unclear. To gain further insights into the correlation between RAB10 and the immune microenvironment, we employed bioinformatics techniques. Nevertheless, additional experimental investigations are required to validate the underlying mechanisms involved in this relationship.

We also predicted the biological functions of RAB10 by GSEA GO enrichment analysis, which revealed that RAB10's biological functions are primarily immune-related, including phagocytosis recognition, complement activation, and immunoglobulin receptor binding. GSEA KEGG analysis suggested that RAB10 may be involved in BC progression through the Hedgehog signaling pathway, Fanconi anemia pathway, Hippo signaling pathway, and other pathways. The results of GSEA GO analysis and immune cell infiltration analysis suggest that RAB10 may be involved in BC progression through immune-related pathways. Although network data are only predictive, easily confounded by statistical modalities, and lack high-level evidence to support them, they can provide new directions for further studies. In the future, we will continue to explore the upstream and downstream genes of RAB10 to further elucidate its mechanism of action.

## Conclusions

Our findings suggest that elevated expression of RAB10 in BC suggests a poor prognosis for BC, and that its ability to promote BC cell proliferation, migration and invasion in vitro is associated with tumor immune infiltrating cells in BC and is a potential biomarker or molecular target for BC.

### Supplementary Information

Supplementary Figure 1.

Supplementary Figure 2.

Supplementary Figure 3.

## Data Availability

The datasets used and/or analysed during the current study are available from the corresponding author upon reasonable request.

## References

[1] Bray F, Ferlay J, Soerjomataram I (2018). Global cancer statistics 2018: GLOBOCAN estimates of incidence and mortality worldwide for 36 cancers in 185 countries. CA A Cancer J. Clin..

[2] Trapani D, Ginsburg O, Fadelu T (2022). Global challenges and policy solutions in breast cancer control. Cancer Treat. Rev..

[3] Turashvili G, Brogi E (2017). Tumor heterogeneity in breast cancer. Front. Med..

[4] Denkert C, Loibl S (2022). Response-based molecular subtyping—emergence of the third generation of breast cancer subtypes. Cancer Cell.

[5] Diekmann Y, Seixas E, Gouw M (2011). Thousands of rab GTPases for the cell biologist. PLoS Comput. Biol..

[6] Yan T, Wang L, Gao J (2018). Rab10 phosphorylation is a prominent pathological feature in Alzheimer’s disease. J Alzheimers Dis..

[7] Chua CEL, Tang BL (2018). Rab 10—a traffic controller in multiple cellular pathways and locations. J. Cell. Physiol..

[8] Wang W, Jia W-D, Hu B, Pan Y-Y (2017). RAB10 overexpression promotes tumor growth and indicates poor prognosis of hepatocellular carcinoma. Oncotarget.

[9] Zhang Y-J, Pan Q, Yu Y, Zhong X-P (2020). microRNA-519d induces autophagy and apoptosis of human hepatocellular carcinoma cells through activation of the AMPK signaling pathway via Rab10. Cancer Manag. Res..

[10] Han H, Shao Q, Liu X (2020). LINC00441 promotes cervical cancer progression by modulating miR-450b-5p/RAB10 axis. Cancer Cell Int..

[11] Zhang X, Wang S, Lin G, Wang D (2020). Down-regulation of circ-PTN suppresses cell proliferation, invasion and glycolysis in glioma by regulating miR-432-5p/RAB10 axis. Neurosci. Lett..

[12] Li Y, Xiong Y, Wang Z (2021). FAM49B promotes breast cancer proliferation, metastasis, and chemoresistance by stabilizing ELAVL1 protein and regulating downstream Rab10/TLR4 pathway. Cancer Cell Int..

[13] Bartha Á, Győrffy B (2021). TNMplot.com: A web tool for the comparison of gene expression in normal, tumor and metastatic tissues. IJMS.

[14] Chandrashekar DS, Karthikeyan SK, Korla PK (2022). UALCAN: An update to the integrated cancer data analysis platform. Neoplasia.

[15] Lánczky A, Győrffy B (2021). Web-based survival analysis tool tailored for medical research (KMplot): Development and implementation. J. Med. Internet Res..

[16] Kanehisa M, Furumichi M, Sato Y (2023). KEGG for taxonomy-based analysis of pathways and genomes. Nucleic Acids Res..

[17] Moasser MM (2007). The oncogene HER2: Its signaling and transforming functions and its role in human cancer pathogenesis. Oncogene.

[18] Lv Q, Meng Z, Yu Y (2016). Molecular mechanisms and translational therapies for human epidermal receptor 2 positive breast cancer. IJMS.

[19] Ben Saad A, Vauthier V, Lapalus M (2021). RAB10 interacts with ABCB4 and regulates its intracellular traffic. IJMS.

[20] Mueller C, Haymond A, Davis JB (2018). Protein biomarkers for subtyping breast cancer and implications for future research. Expert Rev. Proteom..

[21] Drucker A, Yoo BH, Khan IA (2020). Trastuzumab-induced upregulation of a protein set in extracellular vesicles emitted by ErbB2-positive breast cancer cells correlates with their trastuzumab sensitivity. Breast Cancer Res..

[22] Actis Dato V, Chiabrando GA (2021). Activated alpha-2 macroglobulin improves insulin response via LRP1 in lipid-loaded HL-1 cardiomyocytes. IJMS.

[23] Dieci MV, Miglietta F, Guarneri V (2021). Immune Infiltrates in breast cancer: Recent updates and clinical implications. Cells.

[24] Pernas S, Tolaney SM (2022). Clinical trial data and emerging strategies: HER2-positive breast cancer. Breast Cancer Res. Treat..

[25] Choi J, Gyamfi J, Jang H, Seung J (2017). The role of tumor-associated macrophage in breast cancer biology. Histol. Histopathol..

[26] Nelson MA, Ngamcherdtrakul W, Luoh S-W, Yantasee W (2021). Prognostic and therapeutic role of tumor-infiltrating lymphocyte subtypes in breast cancer. Cancer Metastas. Rev..

[27] Muntasell A, Rojo F, Servitja S (2019). NK cell infiltrates and HLA class I expression in primary HER2+ breast cancer predict and uncouple pathological response and disease-free survival. Clin. Cancer Res..

[28] Nazish I, Arber C, Piers TM (2021). Abrogation of LRRK2 dependent Rab10 phosphorylation with TLR4 activation and alterations in evoked cytokine release in immune cells. Neurochem. Int..

[29] Wang D, Lou J, Ouyang C (2010). Ras-related protein Rab10 facilitates TLR4 signaling by promoting replenishment of TLR4 onto the plasma membrane. Proc. Natl. Acad. Sci. U. S. A..

[30] Liu Z, Xu E, Zhao HT (2020). LRRK2 and Rab10 coordinate macropinocytosis to mediate immunological responses in phagocytes. EMBO J..

[31] Tian A-L, Lu M, Zhang F-K (2018). The pervasive effects of recombinant *Fasciola gigantica* Ras-related protein Rab10 on the functions of goat peripheral blood mononuclear cells. Parasites Vectors.

[32] Atashrazm F, Hammond D, Perera G (2019). LRRK2-mediated Rab10 phosphorylation in immune cells from Parkinson’s disease patients. Mov. Disord..

