# Supplementary figures and images for: RAB10 promotes breast cancer proliferation migration and invasion predicting a poor prognosis for breast cancer
Source: Sci Rep. 2023 Sep 14;13:15252. doi: 10.1038/s41598-023-42434-1 (PMC10502149; doi:10.1038/s41598-023-42434-1)

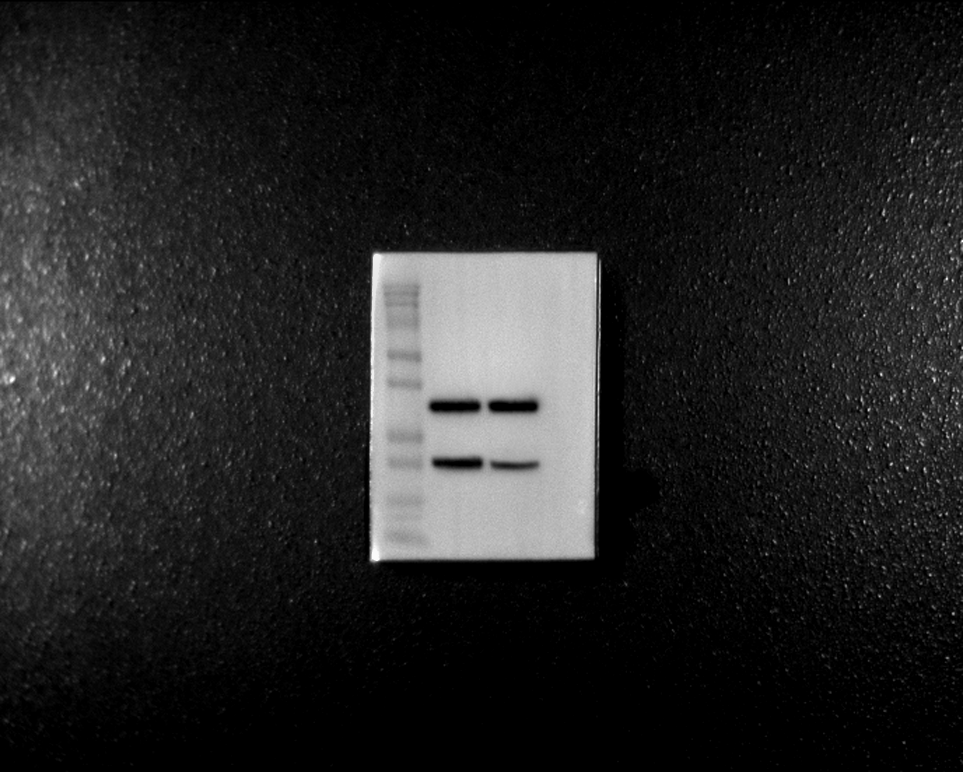

Supplement: Supplementary file 1 — Supplementary Figure 1. [file 41598_2023_42434_MOESM1_ESM.tif]

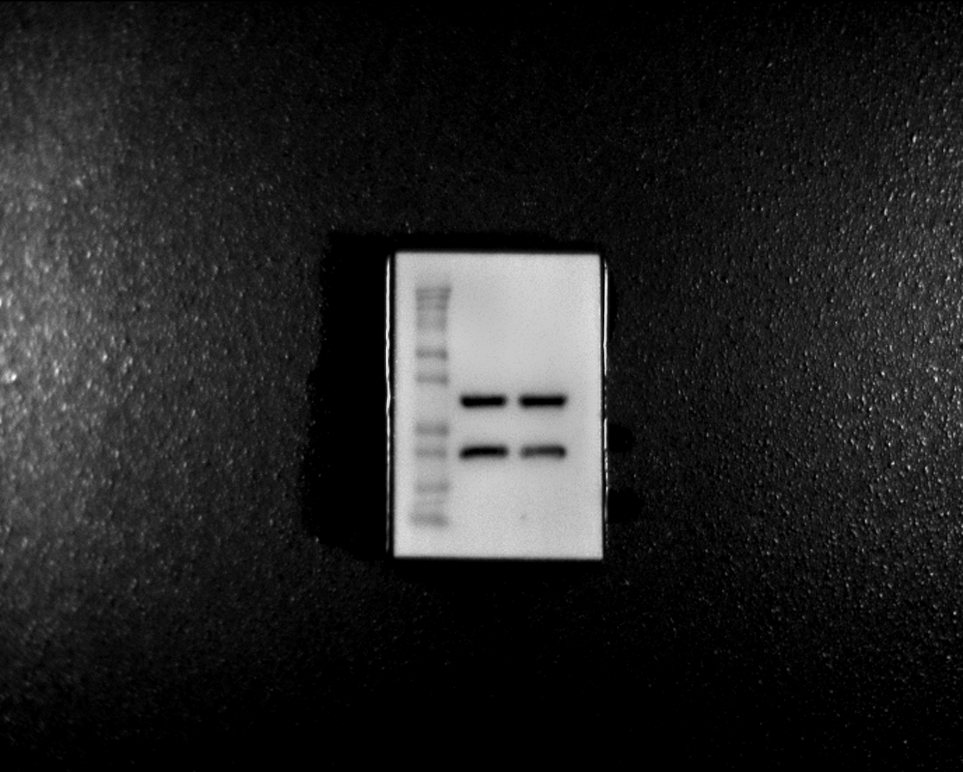

Supplement: Supplementary file 2 — Supplementary Figure 2. [file 41598_2023_42434_MOESM2_ESM.tif]

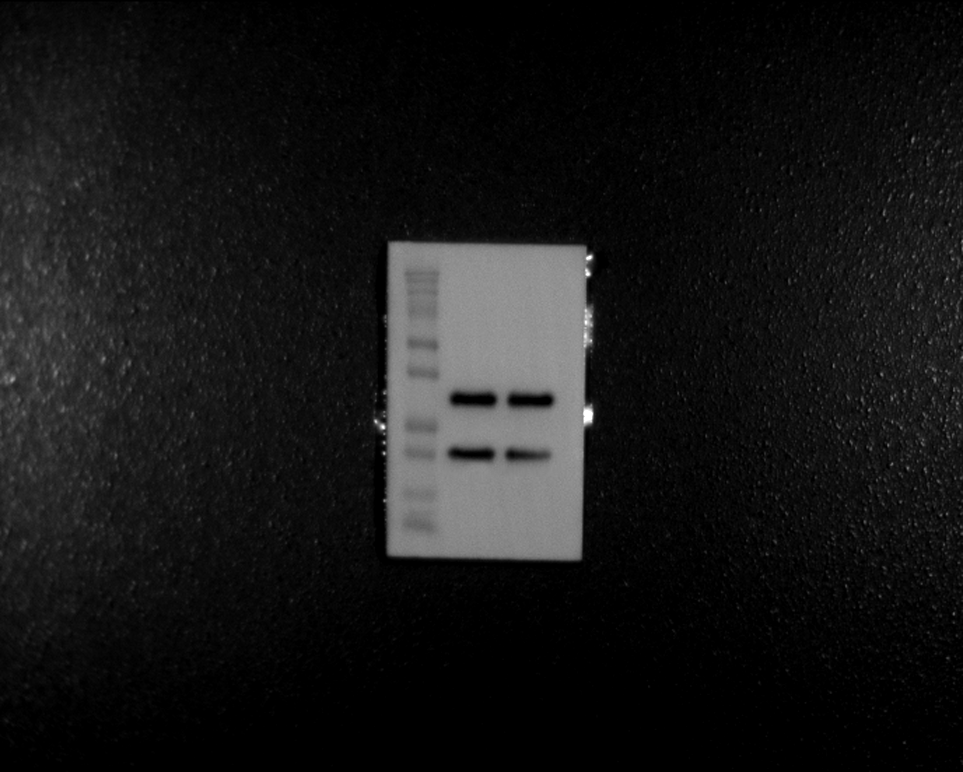

Supplement: Supplementary file 3 — Supplementary Figure 3. [file 41598_2023_42434_MOESM3_ESM.tif]
